# Leptospirosis: A Silent Epidemic Disease

**DOI:** 10.3390/ijerph10127229

**Published:** 2013-12-16

**Authors:** Maria Cristina Schneider, Michel Jancloes, Daniel F. Buss, Sylvain Aldighieri, Eric Bertherat, Patricia Najera, Deise I. Galan, Kara Durski, Marcos A. Espinal

**Affiliations:** 1 Pan American Health Organization, 525 23rd St. NW, Washington, DC 20037, USA; E-Mails: aldighsy@paho.org (S.A.); najerapa@paho.org (P.N.); galand@paho.org (D.I.G.); espinalm@paho.org (M.A.E.); 2 Health and Climate Foundation, 1425 K St. NW Suite 350, Washington, DC 20005, USA; E-Mail: michel.jancloes@gmail.com; 3 Instituto Oswaldo Cruz/Fundação Oswaldo Cruz (Fiocruz), Av. Brasil 4365, Manguinhos, Rio de Janeiro, RJ, CEP 21045-900, Brazil; E-Mail: dbuss@ioc.fiocruz.br; 4 World Health Organization, Avenue Appia 20, Geneva 1211, Switzerland; E-Mails: bertherate@who.int (E.B.); durskik@who.int (K.D.)

This special issue of International Journal of Environmental Research and Public Health is dedicated to leptospirosis, an endemic zoonotic disease that is a cause of many acute undifferentiated fevers, especially in tropical countries [1,2]. While it can be debated whether leptospirosis is an emerging disease, it is evident that it is becoming an emerging public health problem. It is recognized as a disease of epidemic potential that has a significant health impact in many parts of the world. 

Leptospirosis is an excellent example of “One Health”, where the relationship between humans, animals and ecosystems can be used to improve our understanding of this disease and to enhance control strategies [3]. The bacteria *Leptospira interrogans* is pathogenic to humans and animals. It affects a wide variety of animal species, both wild and domestic, which serve as sources of infection for humans [4]. Exposure through water and soil contaminated by the urine of infected animals is the most common route of transmission to people and domestic animals [4].

The burden of leptospirosis is estimated to be 500,000 persons worldwide per year, although new estimations are being developed by an expert consultation group led by the World Health Organization (WHO) [5]. In addition, the number of reported cases associated with natural disasters and flooding have increased with the most notable outbreaks occurring in: Nicaragua (1995), Peru and Ecuador (1998), Orissa (1999), Malaysia (2000), Jakarta (2002), Mumbai (2000 and 2005), and The Philippines (2009) [6,7,8,9,10,11,12]. 

Reviewing the HealthMap database that utilizes different online sources for real-time surveillance of emerging public health threats, there were 787 global alerts for leptospirosis between 2007 and 2013 [13]. More than half of these leptospirosis alerts (63%) occurred in the Americas Region, particularly in Brazil (142 alerts), Nicaragua (45) and Argentina (43) [13]. About ten million people are affected by natural disasters in the Region of the Americas annually, with the majority of them being storms (41%) and floods (35%) [14,15]. However, only half of the countries in this region have reported leptospirosis case surveillance data, which suggests that not all countries have recognized leptospirosis as an important public health threat [16]. The region with the second highest percentage of alerts is the Western Pacific (15%), followed by South-East Asia (14%) and Europe (8%) [13]. The African (1%) and the Eastern Mediterranean Regions (0.5%) do not present many leptospirosis alerts, possibly due to their diagnostic capabilities [13]. 

Leptospirosis cases have been reported in a variety of settings, from large urban centers after floods to remote rural areas with limited access to clean drinking water and sanitation [17,18,19,20]. While it affects mostly vulnerable populations and is often considered a disease of poverty in middle and low income countries; it is also considered an occupational disease affecting rice workers, animal handlers (farmers, veterinarians, butchers), sewer workers, gold mining workers, among others in low, middle and high income countries alike [4,21,22,23]. In recent years, leptospirosis has gained increased attention as it relates to recreational activities among the wildlife and army expeditions [8,24]. The impact on humans may be devastating since the disease can result in hospitalization and time lost from work [25]. In addition, leptospirosis outbreaks pose a burden on health systems and can cause significant economic and social disruption.

Leptospirosis is typically underdiagnosed and underreported. With symptoms ranging from a mild flu-like illness to a more severe and sometimes fatal disease (mortality rate greater than 10%), differentiating leptospirosis from diseases with similar non-specific symptoms such as dengue, malaria and influenza becomes arduous [1,26,27]. Furthermore, laboratory confirmation is difficult as diagnostic tests are expensive and require specific training and equipment, often only found at reference labs. 

Despite these difficulties, leptospirosis remains one of the top ten infectious hazards reported globally in the Event Management System (EMS), the event system that supports the International Health Regulations since its revised version implemented in 2007 [28,29]. In the Americas, leptospirosis events were in the top third, and came only after dengue and influenza as top infectious hazards in the EMS. 

Animal leptospirosis can cause a significant economic impact to local farmers and national economies since it is related to reduced milk production and livestock abortions [30]. Local studies in Central America already indicate the importance of this disease in animals, given that leptospirosis prevalence was found in bovine (31–83%), equine (18–76%), porcine (17–75%) and canine (27–65%), among others animals [31]. Because only a few studies can be found in the literature about the impact of leptospirosis on livestock production, both in small farms and in extensive livestock raising practices, there is an urgent need to have additional studies evaluating its impact and the cost-effective actions required in order to reduce the spread of this disease from animals to humans.

Further, the role that the environment has on leptospirosis outbreaks is not well understood. Recent evidence suggests that climate change may be correlated to the increased number of outbreaks. In addition, research indicates that heavy rains or floods may be drivers for this disease [18,32,33,34,35]. Another possible driver is type of alkaline and neutral soil, which may facilitate longer survival of the bacteria, especially in volcano origin soils [18,30]. Certain ecological conditions may propagate the circulation of peri-domestic rodents and contribute to intensive agriculture production [36]. Also, higher risk of infection has been associated with vulnerable populations living in dense urban or peri-urban areas without waste collection and with inadequate sanitation [33,37]. An increased level of understanding regarding the risk factors could provide information to support countries’ decision makers in identifying risk areas for priority interventions. 

Leptospirosis remains as a neglected disease that suffers from unawareness, despite its increasing number of cases and outbreaks globally [28]. However, the impact this disease has on various sectors, on numerous risk groups, in many countries, and in a variety of settings demonstrates the importance of addressing leptospirosis with a holistic approach in a global perspective. 

This special issue on leptospirosis in the animal-human-ecosystem interface highlights a range of topics, from the complexities surrounding disease transmission between animals, rodents and humans to the need for developing economical preventive and control methods. It demonstrates the importance of developing interdisciplinary, multi-sectorial groups, as the Global Leptospirosis Environmental Action Network (GLEAN), a network which translates research into operational tools to support communities and countries affected by leptospirosis [38]. It brings to light country specific initiatives and research focusing on the socioeconomic factors related to leptospirosis outbreaks and also addresses the impact leptospirosis has on animals. It points out the need for further research on laboratory diagnosis and vaccines for both humans and animals. 

It is evident that an integrated vision within the animal-human-ecosystem interface is necessary in order to orient knowledge about the prediction, detection, prevention and response to outbreaks of leptospirosis. The complex nature of the transmission of leptospirosis and the gap of practical tools to operate at the local level by both human and animal health authorities is a major challenge and it remains for the scientific community to address. We hope this issue will bring to light the many components surrounding leptospirosis as well as promote the need for further research, collaboration, and innovative ideas necessary reduce its global impact. 
